# Effects of gestational age at birth on perinatal structural brain development in healthy term‐born babies

**DOI:** 10.1002/hbm.25743

**Published:** 2021-12-12

**Authors:** Oliver Gale‐Grant, Sunniva Fenn‐Moltu, Lucas G. S. França, Ralica Dimitrova, Daan Christiaens, Lucilio Cordero‐Grande, Andrew Chew, Shona Falconer, Nicholas Harper, Anthony N. Price, Jana Hutter, Emer Hughes, Jonathan O'Muircheartaigh, Mary Rutherford, Serena J. Counsell, Daniel Rueckert, Chiara Nosarti, Joseph V. Hajnal, Grainne McAlonan, Tomoki Arichi, A. David Edwards, Dafnis Batalle

**Affiliations:** ^1^ Department of Forensic and Neurodevelopmental Science, Institute of Psychiatry, Psychology and Neuroscience King's College London London UK; ^2^ Centre for the Developing Brain, School of Imaging Sciences and Biomedical Engineering King's College London London UK; ^3^ MRC Centre for Neurodevelopmental Disorders King's College London London UK; ^4^ Department of Electrical Engineering ESAT/PSI, KU Leuven Leuven Belgium; ^5^ Biomedical Image Technologies, ETSI Telecomunicación Universidad Politécnica de Madrid and CIBER‐BBN Madrid Spain; ^6^ Department of Computing Imperial College London London UK; ^7^ Department of Medicine and Informatics Technical University of Munich Munich Germany; ^8^ Department of Child and Adolescent Psychiatry, Institute of Psychiatry, Psychology and Neuroscience King's College London London UK; ^9^ Paediatric Neurosciences Evelina London Children's Hospital Guy's and St Thomas' NHS Foundation Trust London UK; ^10^ Department of Bioengineering Imperial College London London UK

**Keywords:** neonatal, neurodevelopmental, typical development

## Abstract

Infants born in early term (37–38 weeks gestation) experience slower neurodevelopment than those born at full term (40–41 weeks gestation). While this could be due to higher perinatal morbidity, gestational age at birth may also have a direct effect on the brain. Here we characterise brain volume and white matter correlates of gestational age at birth in healthy term‐born neonates and their relationship to later neurodevelopmental outcome using T2 and diffusion weighted MRI acquired in the neonatal period from a cohort (*n* = 454) of healthy babies born at term age (>37 weeks gestation) and scanned between 1 and 41 days after birth. Images were analysed using tensor‐based morphometry and tract‐based spatial statistics. Neurodevelopment was assessed at age 18 months using the Bayley Scales of Infant and Toddler Development, Third Edition (Bayley‐III). Infants born earlier had higher relative ventricular volume and lower relative brain volume in the deep grey matter, cerebellum and brainstem. Earlier birth was also associated with lower fractional anisotropy, higher mean, axial, and radial diffusivity in major white matter tracts. Gestational age at birth was positively associated with all Bayley‐III subscales at age 18 months. Regression models predicting outcome from gestational age at birth were significantly improved after adding neuroimaging features associated with gestational age at birth. This work adds to the body of evidence of the impact of early term birth and highlights the importance of considering the effect of gestational age at birth in future neuroimaging studies including term‐born babies.

## Graphical abstract

Babies born in the early term period (37–38 weeks) have poorer neurodevelopment than those born at full term (40–41 weeks). We demonstrate neonatal MRI correlates of gestational age at birth in healthy term‐born babies, and their relationship to neurodevelopment at age 18 months. We find effects of gestational age at birth on brain structure, white matter microstructure, and later neurodevelopment.

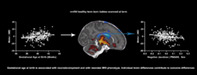

## 1 INTRODUCTION

Study of inter‐individual variation in the duration of pregnancy has taken place for at least 2,500 years (Malamitsi‐Puchner, 2017). Academic consensus for the vast majority of this time has been that childbirth after 37 weeks gestational age (usually defined as the time since the first day of the mother's last menstrual period) is optimal, and infants born earlier than this are at an increased risk of negative outcomes (Budin, 1907). Recent research has challenged the idea of a term—preterm binary classification and proposed that babies born earlier within the 37 to 41‐week (term) range may experience suboptimal early life development. The first large study to demonstrate this, published in 2012, used a cohort of 18,818 infants and found that individuals born between 37 and 38 weeks gestational age had a higher risk of adverse health and developmental outcomes at age 3 compared to those born between 39 and 41 weeks gestational age (Boyle et al., 2012). Subsequent studies have linked other adversities with early term birth, including higher risks of neonatal (Murzakanova et al., 2020) and later childhood illness (Coathup et al., 2020) and less favourable neurodevelopmental outcomes in early life (Hua et al., 2019; Rose et al., 2013).

The underlying mechanism of these differences is not fully understood, although several hypotheses have been proposed. Brain development occurs rapidly in the third trimester—being born (and hence removed from the supportive maternal environment) earlier in this period may disrupt normal neurodevelopment (Davis & Sandman, 2010). Outcome differences could also be due to perinatal risk factors: infants born earlier have lower birthweights and a higher risk of perinatal morbidity (Murzakanova et al., 2020), which may lead to worse childhood neurodevelopmental outcomes (Doctor, O'Riordan, Kirchner, Shah, & Hack, 2001; Taylor, Minich, Bangert, Filipek, & Hack, 2004). In many countries mothers who experience complications during pregnancy which themselves may adversely affect the baby, such as pre‐eclampsia or intrauterine growth restriction, often have labour medically induced at a gestational age of 37 weeks, which could also contribute to differences in perinatal risk and on average poorer outcomes in early term born infants (Coates et al., 2020).

A small number of studies have examined imaging correlates of gestational age at birth (GAAB) in term‐born babies, all exclusively using diffusion tensor imaging acquired in the early neonatal period (Broekman et al., 2014; Jin et al., 2019; Ou et al., 2017). These studies all report similar results, namely lower fractional anisotropy (FA) and higher mean diffusivity (MD) in infants born earlier in the term period. Associations of GAAB with MRI features in term only populations have also been reported in later childhood, including lower total brain volume in a cohort of 10‐year old children (El Marroun et al., 2020), and a positive association of GAAB with functional connectivity in a cohort of 6–10 year olds (Kim et al., 2014).

One likely reason for the paucity of studies demonstrating a brain effect of GAAB in the neonatal period is that the majority of neonatal MRI studies thus far have focused on preterm or clinical risk populations (Batalle, Edwards, & O'Muircheartaigh, 2018). However, this limitation can be overcome with the developing Human Connectome Project (dHCP) data which consists of high quality neonatal MRI data and complementary neurodevelopmental outcomes at age 18 months from a large cohort of healthy term‐born individuals (Hughes et al., 2017), allowing us to test the hypothesis that GAAB will be correlated with neonatal brain structure in term‐born babies (independently of age at scan). To do so, we analysed the volumetric (using tensor‐based morphometry [TBM]) and white matter (using tract based spatial statistics [TBSS]) brain correlates of GAAB and their association with neurodevelopmental outcomes in a sample of 454 term‐born neonates.

## 2 METHODS

### 2.1 Sample

This study is based on a prospective sample of neonates participating in the dHCP (http://www.developingconnectome.org/). Data collection for the project took place in London, UK from 2015 to 2020. This project has received UK NHS research ethics committee approval (14/LO/1169, IRAS 138070), and written informed consent was obtained from parents.

For the purposes of this study, we have included only healthy term‐born singletons. Exclusion criteria were as follows: GAAB <37 weeks, admission to neonatal intensive care following birth, non‐singleton birth, medical complication reported during pregnancy by mother, intrauterine growth restriction or small for gestational age birthweight, or a visible abnormality of possible clinical or analytical significance on MRI following reporting by an experienced Paediatric Neuroradiologist.

### 2.2 Image acquisition

Neonatal MRI data were acquired on a Phillips 3 Tesla Achieva system (Philips Medical Systems, Best, The Netherlands) at the Evelina Newborn Imaging Centre, Evelina London Children's Hospital. All infants were scanned during natural sleep without sedation as previously described (Hughes et al., 2017), including a bespoke transport system, positioning device and an optimally sized neonatal 32‐channel receive coil with a custom‐made acoustic hood. All scans were supervised by a neonatal nurse and/or paediatrician who monitored heart rate, oxygen saturation, and temperature throughout the scan.

T2‐weighted images were obtained using a Turbo Spin Echo sequence, acquired in two stacks of 2D slices (in sagittal and axial planes), using parameters: TR = 12 s, TE = 156 ms, SENSE factor 2.11 (axial) and 2.58 (sagittal), acquisition resolution 0.8 × 0.8 × 1.6 mm^3^ with 0.8 mm slice overlap, reconstructed to 0.8 mm^3^ isotropic resolution, and diffusion images were acquired using parameters TR = 3,800 ms; TE = 90 ms; SENSE factor = 1.2; multiband factor = 4; acquisition resolution 1.5 × 1.5 × 3.0 mm^3^ with 1.5 mm slice overlap. Diffusion gradient encoding included images collected at *b* = 0 s/mm^2^ (20 repeats), *b* = 400 s/mm^2^ (64 directions); *b* = 1,000 s/mm^2^ (88 directions), *b* = 2,600 s/mm^2^ (128 directions) (Hutter et al., 2018), and images were reconstructed to a final resolution of 1.5 × 1.5 × 1.5 mm^3^ (Christiaens et al., 2021; Hutter et al., 2018).

### 2.3 Image processing

Motion‐correction and slice‐to‐volume reconstruction of T2 weighted images were carried out using a dedicated algorithm as previously described (Cordero‐Grande, Hughes, Hutter, Price, & Hajnal, 2018). These images were subsequently registered to a dHCP week‐specific template (according to each individual's postmenstrual age at scan) in a common space corresponding to gestational age of 40 weeks (Schuh et al., 2018) using the symmetric normalisation (SyN) algorithm from Advanced Normalisation Tools (ANTs), version 3.0 (Avants & Gee, 2004). Affine and non‐linear transformations were performed, with the last of these used to create deformation tensor fields in template space to remove the effect of individual variations in head size from the final analysis. The resulting tensor fields were used to calculate scalar Jacobian determinants, which were subject to logarithmic transformation, using ANTs. Log‐Jacobian determinant maps (hitherto referred to as Jacobians) were smoothed with a sigma of 3.5 mm full width at half maximum Gaussian filter. Images were finally resized to an isotropic voxel size of 1 mm prior to TBM analysis (Ashburner & Friston, 2000). The anatomical location of areas of significance was determined using the dHCP atlas (Makropoulos et al., 2018).

Diffusion MRI was pre‐processed as previously described (Kelly et al., 2019). Briefly, images were denoised (Cordero‐Grande et al., 2018), Gibbs ringing suppressed (Kellner, Dhital, Kiselev, & Reisert, 2016) and reconstructed using a slice‐to‐volume motion correction technique that uses a bespoke spherical harmonics and radial decomposition method, together with outlier rejection, distortion and slice profile correction (Christiaens et al., 2021). Tensors were reconstructed and non‐linearly registered to a population‐based FA template using DTI‐TK (Zhang, Yushkevich, Alexander, & Gee, 2006). Mean diffusivity (MD), axial diffusivity (AD), radial diffusivity (RD), and FA maps for each subject were subsequently generated in template space, and re‐integrated into the FSL TBSS pipeline (thresholded at 0.15) (Ball et al., 2010; Jenkinson, Beckmann, Behrens, Woolrich, & Smith, 2012). The anatomical locations of areas of significance in TBSS were determined using the JHU neonatal atlas (Oishi et al., 2011). This was registered directly to the population‐based FA template using ANTs (Avants & Gee, 2004).

### 2.4 Follow‐up

Neurodevelopmental assessment was offered to all participants at 18 months of age. Neurodevelopmental performance was assessed using the Bayley Scales of Infant and Toddler Development, Third Edition (Bayley‐III; Bayley, 2006). Assessments were performed at St Thomas' Hospital, London by two experienced assessors (authors A. C., a paediatrician and S. F., a chartered psychologist). The cognitive, motor and language composite scores are used for analyses in this study. Index of multiple deprivation (IMD) (a geographically defined composite social risk score comprising data on income, employment, health, education, living environment, and crime) was calculated from the mother's home address at the time of birth and included as a covariate in all associations with follow‐up outcome data (Abel, Barclay, & Payne, 2016).

### 2.5 Statistical analysis

TBM and TBSS analyses were performed using the *randomise* tool for nonparametric permutation inference in FSL with 10,000 permutations per test (Winkler, Ridgway, Webster, Smith, & Nichols, 2014). Threshold free cluster enhancement and family wise error rate were applied to correct for voxel‐wise multiple comparisons. All general linear models contained postmenstrual age at scan and sex as covariates.

Associations between GAAB, MRI findings and Bayley‐III scores were performed using univariate linear regression in SPSS statistics 26. Block design models were used to assess the influence of MRI data on associations between GAAB and neurodevelopment. To compare blocks, the significance of the change in *r* ^2^ at each successive block in the model was tested. Graphical figures were drawn in GraphPad Prism 8.

Specifics of tests used for associations of GAAB and neurodevelopmental outcome are indicated with each result.

## 3 RESULTS

### 3.1 Population

At the time of the study commencing, MRI data had been collected and pre‐processed from 771 individuals. Then, 251 individuals were excluded due to being born preterm, 12 due to non‐singleton births, 18 due to medical complications of pregnancy, 26 due to a birthweight <10th centile (small for gestational age), and 10 due to visible abnormalities of possible clinical significance on MRI. This left 454 individuals included in the study. Of the 454 individuals, 374 had diffusion MRI data, 332 had T2 data and were followed up at 18 months, and 281 had T2 data, DWI data, and were followed up at 18 months. The demographic details of the cohort are summarised in Table 1.

**TABLE 1:** Demographic details of cohort

	T2 MRI	Diffusion MRI	Follow‐up and T2 MRI	Follow‐up and both MRI modalities
*N*	454	374	332	281
Male	247	204	179	146
Female	207	170	153	135
Gestational age at birth [weeks]	40.1 (39.1–41.0)	40.1 (39.3–40.7)	40.1 (39.1–40.9)	40.1 (39.4–41.0)
Post‐menstrual age at scan [weeks]	41.3 (40.3–42.6)	40.9 (40.3–42.0)	41.2 (40.1–42.4)	41.0 (40.4–42.1)
APGAR (5 min)	9 (8–10)	9 (7–10)	9 (8–10)	9 (8–10)
Birthweight centile	52 (28–77)	51 (29–76)	57 (39–79)	51 (29–77)
Age at follow‐up [months]			18.3 (18.0–18.7)	18.3 (18.0–18.8)
Bayley‐III cognitive			100 [95–110]	100 [95–105]
Bayley‐III motor			97 [89–107]	97 [89–107]
Bayley‐III language			103 [97–107]	103 [97–110]

*Note*: Median (IQR) indicated.

Abbreviation: Bayley‐III, Bayley Scales of Infant and Toddler Development, Third Edition.

### 3.2 Brain volume (TBM)

**FIGURE 1:**
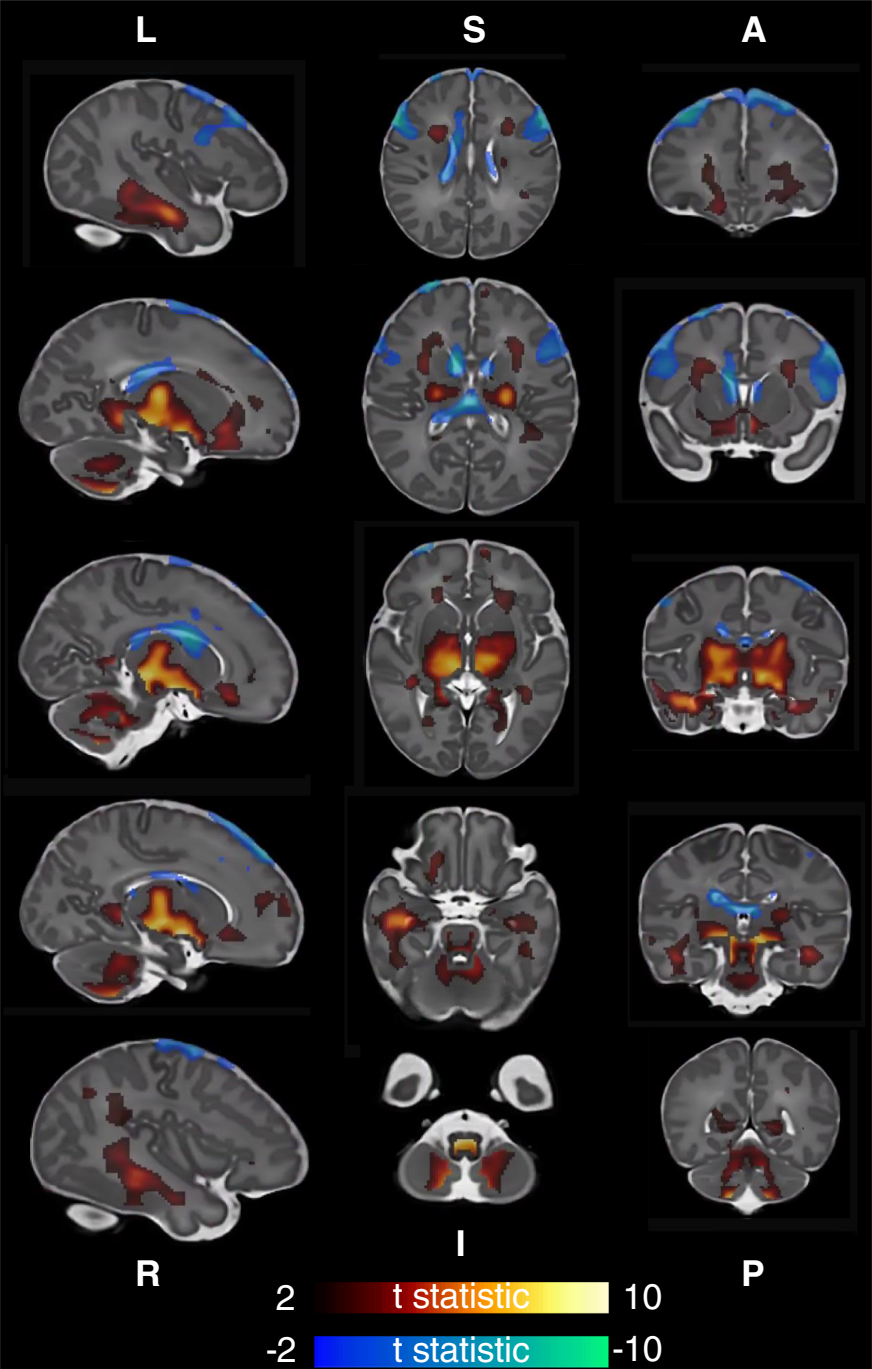
Association of gestational age at birth with brain volume. T statistic of areas of significant (*p* < .025) correlation shown—positive in red‐yellow, negative in blue‐green. First column left to right, second column superior to inferior, and third column anterior to posterior. Analysis corrected for postmenstrual age at scan and sex

Voxel‐wise analysis of relative brain volume using TBM showed a positive correlation of GAAB (corrected for postmenstrual age at scan and sex) with relative brain volume (Jacobians) in the deep grey matter (bilaterally in the lentiform nucleus and thalamus), cerebellum, inferior parts of the frontal and temporal lobes, and parts of the brainstem bilaterally. The relative volumes of parts of the ventricles were negatively correlated with GAAB (Figure 1).

### 3.3 White matter diffusion measures (TBSS)

**FIGURE 2:**
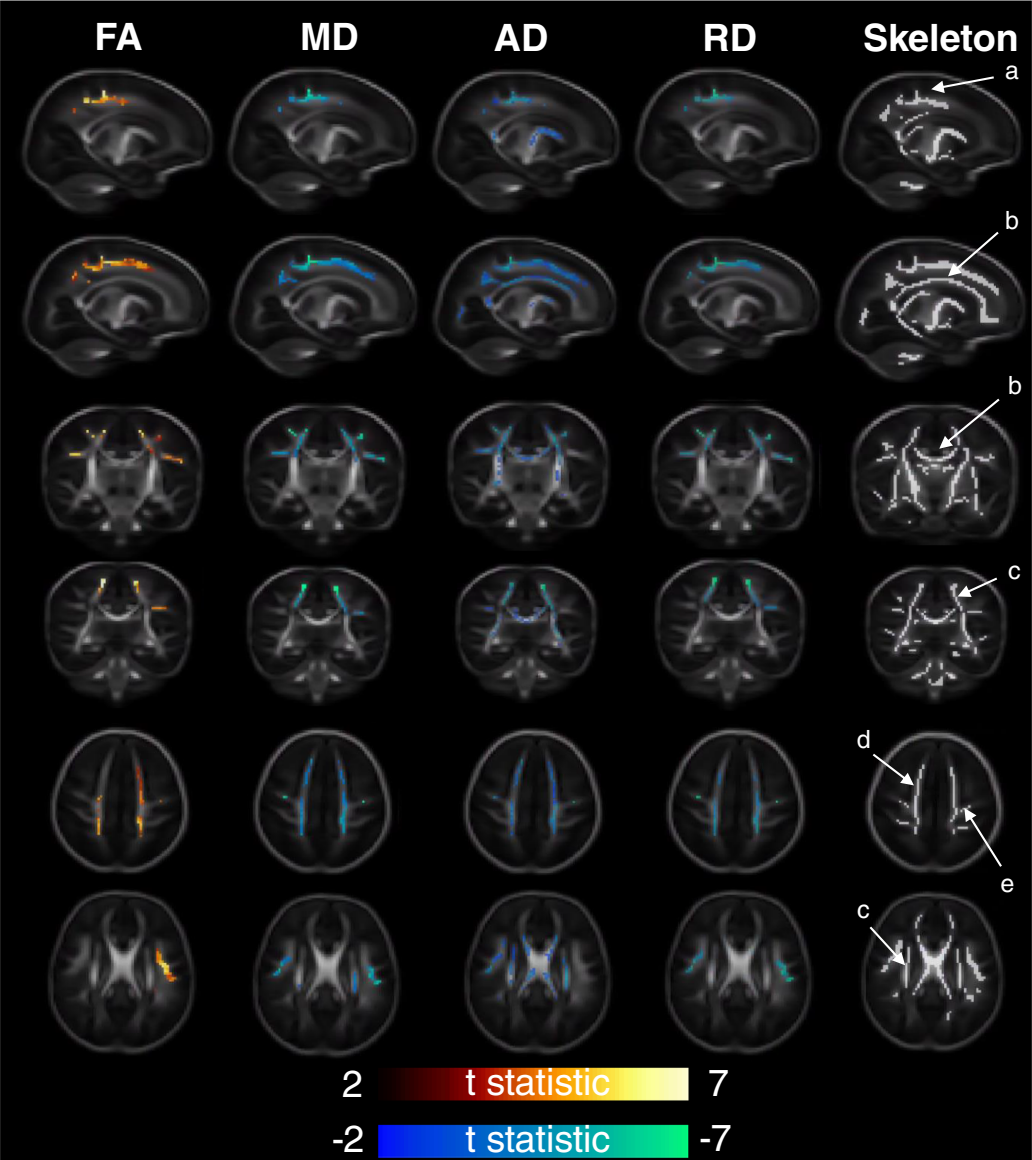
Association of gestational age at birth with white matter diffusivity measures. T statistic of areas of significant (*p* < .025) correlation shown—positive in red‐yellow, negative in blue‐green. From left to right—Fractional anisotropy (FA), mean diffusivity (MD), axial diffusivity (AD), radial diffusivity (RD), and representative diffusion skeleton. From top to bottom—Axial (superior), axial (inferior), sagittal (L), sagittal (R), coronal (anterior), and coronal (posterior). Analysis corrected for post‐menstrual age at scan and sex. Labelled skeleton tracts based on Oishi et al. (2011): (a) cingular gyrus, (b) corpus callosum, (c) superior corona radiata, (d) projection fibres to superior fontal gyrus, and (e) projection fibres to precentral gyrus

Analysis of white matter diffusion MRI characteristics using TBSS showed that gestational age at birth was positively correlated with FA and negatively correlated with MD, AD, and RD. AD was most strongly associated with GAAB, and FA least strongly associated. Parts of the superior corona radiata and projection fibres to the precentral gyrus bilaterally were significantly associated with GAAB in all DTI modalities. The MD, AD, and RD of superior longitudinal fasciculus bilaterally were significantly associated with GAAB, although FA was not significant in this tract. The AD and RD of the corona radiata bilaterally were also significantly associated with GAAB, as was the AD of the corpus callosum (Figure 2).

### 3.4 Neurodevelopment at 18 months

**FIGURE 3:**
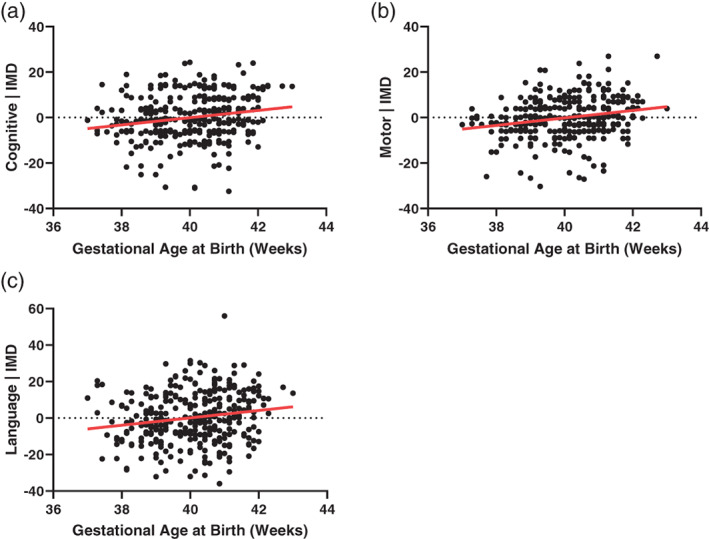
Association of gestational age at birth with neurodevelopment at 18 months (*n* = 332). (a–c) Bayley Scales of Infant and Toddler Development, Third Edition (Bayley‐III) ((a) cognitive adj. *r* ^2^ = .04, *p* = .002, (b) motor adj. *r* ^2^ = .04, *p* = .001, (c) language adj. *r* ^2^ = .04, *p* = .001). All analyses corrected for index of multiple deprivation (IMD) score

GAAB was positively correlated with all sub‐domains of the Bayley‐III Scales (Cognitive adj. *r* ^2^ = .04, *p* = .002, Motor adj. *r* ^2^ = .04, *p* = .001, Language adj. *r* ^2^ = .04, *p* = .001) (Figure 3). For every 1‐week increase in GAAB, the increase in adjusted Bayley‐III scores were cognitive 1.56, motor 1.42, and language 2.38.

**FIGURE 4:**
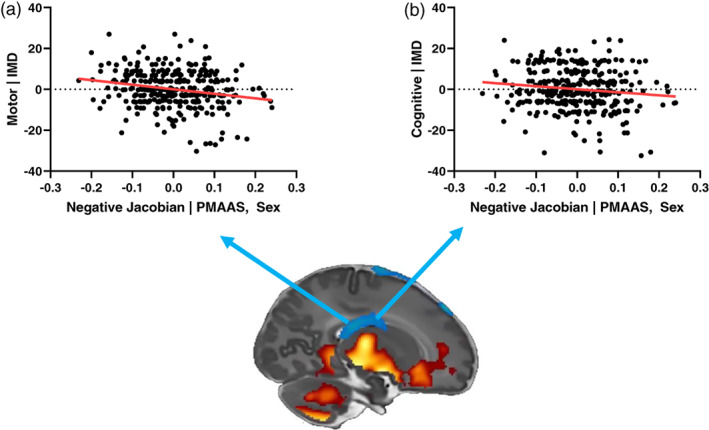
Association of regional volumes with neurodevelopment at 18 months (*n* = 332). Regional volume negatively correlated with gestational age at birth associated with (a) Bayley Scales of Infant and Toddler Development, Third Edition (Bayley‐III) motor score (adj. *r* ^2^ = .04, *p* < .001) and (b) Bayley‐III cognitive score (adj. *r* ^2^ = .02, *p* = .02). Coloured arrows on sagittal section indicate region used in each analysis. Bayley‐III scales corrected for IMD score, mean regional volume feature values corrected for post‐menstrual age at scan (PMAAS) and sex

An exploratory analysis of the influence of the inter‐individual brain differences shown in Figures 1 and 2 on individual outcome differences was subsequently performed. For each neuroimaging modality, the mean feature value of the region significantly associated with gestational age at birth was extracted. This gave six feature values per subject (volume positively associated with GAAB, volume negatively associated with GAAB, FA, MD, AD, and RD associated with GAAB). The association of each of these values with the Bayley‐III neurodevelopmental subscales was assessed using linear regression. As there are three subscales (cognitive, motor, language) this meant that 18 total analyses were performed. Then, 16/18 analyses found no significant association, and 2 were significant, although only one (volume negatively associated with GAAB—Bayley‐III Motor Composite Score) would remain significant after correction for multiple comparisons (Figure 4, Supplementary Table 1).

Next, the mean feature values were added to each of the linear regression analysis of GAAB and Bayley‐III outcome shown in Figure 4 (along with the neuroimaging covariates post‐menstrual age at scan and sex). Feature values were added in two groups—volume features and diffusion features. Analyses of the effect of adding brain volume information were performed in individuals who had a T2 scan and follow‐up (*n* = 332), and analyses of adding brain volume and white matter diffusion information were performed in individuals who had a T2 scan, a DWI scan and follow‐up (*n* = 281). Adding volume significantly improved all models (the F statistic of the model with imaging data added is significantly greater (*p* < .05) than the model without imaging data), whereas adding diffusion features only improved the language model (Table 2).

**TABLE 2:** Modulation of gestational age at birth—outcome association with neuroimaging findings. Analyses performed using univariate linear regression with separate models for cognitive (a), motor (b), and language (c) composite scores from the Bayley‐III scales. The basic model compares gestational age at birth and outcome with IMD as a covariate. The volume model adds the mean feature value (Jacobians) of regions positively or negatively associated with gestational age at birth (shown in Figure 1), as well as the imaging covariates post‐menstrual age at scan and sex, and the DTI model adds the mean feature value of FA, MD, AD, and RD regions associated with gestational age at birth (shown in Figure 2), as well as the imaging covariates post‐menstrual age at scan and sex. Analysis of the effect of the volume model assessed in all individuals who had a T2 scan and follow‐up (*n* = 332), analysis of the effect of the DTI and DTI + volume models additionally assessed in individuals who had a T2 scan, a DWI scan, and follow‐up (*n* = 281)

	*df*	Adjusted *r* ^2^	Model *p*‐value	F change from GAAB + IMD	F change *p*‐value
(a) Cognitive					
*n* = 332					
Y = GAAB + IMD	2	.038	.002	—	—
Y = GAAB + IMD + [volume]	6	.063	.001	2.82	.025
*n* = 281					
Y = GAAB + IMD	2	.046	.001	—	—
Y = GAAB + IMD + [volume]	6	.086	<.001	4.08	.003
Y = GAAB + IMD + [DTI]	8	.046	.008	1.00	.423
Y = GAAB + IMD + [volume] + [DTI]	10	.078	<.001	2.23	.026
(b) Motor					
*n* = 332					
Y = GAAB + IMD	2	.042	.002	—	—
Y = GAAB + IMD + [volume]	6	.080	<.001	4.40	.002
*n* = 281					
Y = GAAB + IMD	2	.024	.012	—	—
Y = GAAB + IMD + [volume]	6	.059	.001	3.51	.008
Y = GAAB + IMD + [DTI]	8	.031	.037	1.30	.258
Y = GAAB + IMD + [volume] + [DTI]	10	.053	.006	2.06	.041
(c) Language					
*n* = 332					
Y = GAAB + IMD	2	.044	.001	—	—
Y = GAAB + IMD + [volume]	6	.083	<.001	3.93	.004
*n* = 281					
Y = GAAB + IMD	2	.048	<.001	—	—
Y = GAAB + IMD + [volume]	6	.089	<.001	4.14	.003
Y = GAAB + IMD + [DTI]	8	.082	<.001	3.05	.011
Y = GAAB + IMD + [volume] + [DTI]	10	.090	<.001	2.85	.007

*Note*: [Volume] = mean feature value (Jacobians) of regions positively associated with gestational age at birth, mean feature value of regions negatively associated with gestational age at birth, post‐menstrual age at scan, and sex. [DTI] = mean feature value of FA regions positively associated with gestational age at birth, mean feature value of MD, AD, and RD regions negatively associated with gestational age at birth, post‐menstrual age at scan, sex.

Abbreviations: AD, axial diffusivity; Bayley‐III, Bayley Scales of Infant and Toddler Development, Third Edition; DTI, diffusion tensor imaging; FA, fractional anisotropy; GAAB, gestational age at birth; IMD, index of multiple deprivation; MD, mean diffusivity; RD, radial diffusivity.

## 4 DISCUSSION

Gestational age at birth related MRI findings were observed in a cohort of healthy term‐born babies. Lower gestational age at birth was also associated with poorer neurodevelopment, and individual differences in neurodevelopment in our cohort may be partially explained by neonatal brain biology.

Infants born earlier had smaller deep grey matter volumes, smaller cerebellar volumes, and relatively larger ventricular volume (Figure 1). This anatomical pattern is similar to that observed in babies born late preterm (Gui et al., 2019). The pattern of white matter microstructural change seen here in infants born earlier (lower FA, higher MD, AD, and RD) is also similar to that observed in late preterm infants compared to term controls (Kim, Park, Kim, Hwang, & Lee, 2016), although the anatomical distribution of areas of significant association with GAAB in our cohort (in which the corona radiata and it's projections appear most affected) is somewhat different to those seen in late preterm babies, where the corpus callosum is by far the most commonly implicated tract (Dibble, Ang, Mariga, Molloy, & Bokde, 2021). In addition to increasing antenatally, the FA of major white matter tracts continues to increase in the first years of life, while MD, AD, and RD decrease (Geng et al., 2012; Loh et al., 2012). The pattern of GAAB‐related DTI findings observed could be conceptualised as slower development in babies born at younger ages. In our cohort, FA was comparatively less influenced by GAAB than MD, AD, or RD. At the time of scan acquisition in this cohort, myelination is incomplete across the brain. Processes resulting in an overall reduction in free water in major white matter tracts (such as membrane permeability reduction and oligodendrocyte proliferation) are ongoing, and these may have a greater influence on MD than FA (Pecheva et al., 2018).

In addition to the neuroimaging results, we demonstrate a GAAB association with neurodevelopmental outcome at 18 months, as assessed with the Bayley‐III scales. This finding adds to a body of literature demonstrating poorer average performance on cognitive tests in individuals born in the early term period (Chan, Leong, Malouf, & Quigley, 2016). Our results are broadly in keeping with one previous study which demonstrated a linear association between GAAB and Bayley‐II sub‐scales at 12 months in a cohort of 232 infants from Southern California, reporting an *r* ^2^ value of .12 for the Mental Development Index and .08 for the Psychomotor Development Index (Espel, Glynn, Sandman, & Davis, 2014).

We build upon these studies by demonstrating how inter‐individual brain differences may also affect later neurodevelopmental outcome. Two direct imaging—outcome associations were observed: the volume of brain regions which negatively correlated with GAAB (anatomically comprised of parts of the ventricles and a small region of cerebrospinal fluid) also negatively correlated with Bayley‐III motor (adj. *r* ^2^ = .04, *p* < .001) and cognitive score (adj. *r* ^2^ = .02, *p* = .02), although the latter of these would not survive multiple comparisons correction (Figure 4). There was no direct association between any DTI metric and Bayley‐III scores. It is worth noting that the amount of Bayley‐III score variance explained by brain volume regions of interest was less than that explained by gestational age at birth alone. These results are novel but should be viewed with a degree of caution, the regions of interest used were of course defined by their association with GAAB in this specific cohort, which itself correlates with Bayley‐III scores (Figure 3). Adding mean feature values of the relative volume (Jacobians) in these regions (chiefly the basal ganglia, parts of the cerebellum and brain stem, and ventricles) significantly improved all linear regression models of GAAB‐outcome, although adding mean feature values of white matter regions significantly associated with GAAB (of which the most important anatomical location is the corona radiata and it's projections) only improved the model of language (Table 2).

Individual volume differences appear to be more important in future neurodevelopmental outcome than diffusion differences in this cohort. This may be due to the different temporal trajectories of cortical structural development and major white matter tract myelination (Sampaio & Truwit, 2001). Relative brain volume changes observed in the foetal or early neonatal period, including the volumes of the ventricles, cerebellum, and basal ganglia (as seen in our cohort), have previously been linked to later neurodevelopmental outcome in several other cohorts (Alarcon et al., 2013; Donald et al., 2016; Hahner et al., 2019; Mirmiran et al., 2004; Van Kooij et al., 2012), with the same direction of association observed as in our cohort (larger ventricles correlating with less favourable outcomes, larger cerebellum and basal ganglia correlating with more favourable outcomes). Although we did not find any direct association between any DTI measure and neurodevelopment the diffusivity of some of the regions associated with GAAB in our study have previously been linked with neurodevelopment in early life in other cohorts; including the corticospinal tract (Tusor et al., 2017), cingulum (Lee et al., 2021), and corona radiata (Parikh et al., 2021), again with the same direction of association as observed with GAAB in our study (lower FA, higher MD, AD, or RD correlate to less favourable outcome). A limitation in direct comparison with our work is that the populations in these studies were either preterm or had an established clinical risk. This is a general trend in the neonatal MRI‐neurodevelopment literature—other studies use populations that have a pre‐existing increased risk of a negative outcome, such as punctate lesions (Hayman et al., 2019), neonatal stroke (Dinomais et al., 2015), neonatal critical illness (Ramadan, Paul, Morton, Peacock, & Greenough, 2012), or high parental age (Gale‐Grant et al., 2020). One study using a mixed cohort of term‐ and preterm‐born babies identified limited associations between major white matter tract diffusion measures in the neonatal period and cognitive function at age 1, although notably did not find any association with cognitive function at age 2 (Girault et al., 2019). We have found no association between major white matter tract diffusion measures in the neonatal period and neurodevelopment at age 18 months, which is broadly in keeping with these findings.

Our study has some limitations. The most significant of these is the mathematical difficulty of delineating the effect of GAAB on MRI appearance with the much more dominant effect of brain maturation over time, as measured by postmenstrual age at scan. In a cohort of term‐born babies scanned as soon as possible postnatally, these two variables are colinear (as is the case in our cohort, Supplementary Figure 1). Some of the regions whose volume positively correlates with GAAB also negatively correlate with gestational age at scan, and vice versa: for example, the relative volume of the basal ganglia and brainstem is positively correlated with GAAB and negatively correlated with postmenstrual age at scan (Figure 1, Supplementary Figure 2). Ideally, to isolate the effect of GAAB, all participants would be scanned at the same postmenstrual age—however no current such dataset exists. During the immediate postnatal period, rapid myelination is a further potential cause of post‐menstrual age related artefact—boundary‐based registration methods (as we have employed here) may result in over estimation of Jacobian values as post‐menstrual age at scan increases (Oishi, Chang, & Huang, 2019). However, it is unlikely that over correction of brain maturation or registration artefact accounts for all of the GAAB associated brain volume differences seen here as the volumes of the ventricles (negatively associated with GAAB) were not associated with post‐menstrual age at scan, and the volume of parts of the cerebellum were positively associated with both GAAB and post‐menstrual age at scan. The areas of the white matter skeleton whose FA is significantly positively associated with GAAB are also significantly positively associated with post‐menstrual age at scan, and the areas of MD, AD, and RD significantly negatively associated with GAAB are significantly negatively associated with post‐menstrual age at scan. A final limitation to be aware of is that some individuals in this cohort scored <85 on Bayley‐III at age 18 months. In the absence of any risk factor for aberrant neurodevelopment, we have elected not to exclude these individuals from our analyses. The dHCP dataset contains comparatively little information regarding the period between birth and age 18 months, and it may be the case that adverse medical or social events have occurred for some of our participants prior to follow‐up. We have used an area‐based summary score of socioeconomic deprivation (the IMD score) to adjust outcomes, and have considered the use of other covariates in our models (Supplementary Figure 4); however, there are of course multiple other well evidenced influences on neurodevelopment that are unaccounted for, such as details of breastfeeding status (Eickmann et al., 2007), parental education (Ross & Perlman, 2019), and parental mental health (Neamah et al., 2018).

At the time of writing, all previously published studies which associated MRI findings in the neonatal period with GAAB (Broekman et al., 2014; Jin et al., 2019; Ou et al., 2017) use postnatal days of life as a covariate rather than post‐menstrual age at scan. This likely gives results which are more representative of general maturation than of the effect of GAAB. This phenomenon can be illustrated by repeating our analysis of the association of GAAB and brain volume with postnatal age as a covariate in the place of post‐menstrual age at scan (Supplementary Figure 3). The results are very similar to the effect of post‐menstrual age (Supplementary Figure 2) but are not similar to the effect of GAAB (Figure 1).

Our study has several strengths. We have used a large homogenous cohort, which improves the quality of neuroimaging analysis and removes potential bias from abnormally low birthweights and medical complications in early term but does limit clinical translatability of our findings. We have used well established MRI analysis methods. An immediate avenue for future research would be to investigate the effect of GAAB on functional connectivity measures in the neonatal period as these have been shown to be significant in older children (Kim et al., 2014).

These results are important for future neuroimaging studies. Neonatal MRI is now well established within the neuroimaging field, and an increasing number of studies focus on term‐born infants. Many of these do not include GAAB as a covariate in analyses (Feldmann et al., 2020; Glass et al., 2021; Merhar et al., 2020)—this study firmly suggests that analyses of MRI in term‐born cohorts should be GAAB corrected. The results are also interesting from a clinical perspective, although it is important to note that the homogeneous sample used here is not representative of a typical population, and equally important to note that the absolute magnitude of GAAB associated Bayley‐III differences is small. Currently 37 weeks gestational age is viewed as a cut‐off at which labour may be optimally induced if there is an indication to do so—8% of all births in the United States in 2006 were induced in early term (Murthy, Grobman, Lee, & Holl, 2011). Caesarean section is also routinely electively performed before 40 weeks—guidelines in both the United Kingdom and the United States recommend any time from 39 weeks, although both cite perioperative morbidity rather than any concern about infant neurodevelopment as their rationale for this gestational age (The American College of Obstetricians and Gynecologists, 2019; The National Institute for Health and Care Excellence, 2011). Our results add to a body of literature demonstrating specific correlates of early term birth which merit further investigation.

This work advances our understanding of the neurodevelopmental effects of birth at different gestational ages after 37 weeks. The finding of significant associations of GAAB with MRI soon after birth is important to consider in neonatal neuroimaging studies.

## Supporting information

Effects of gestational age at birth on perinatal structural brain development in healthy term born babies: Supplementary Material

## Data Availability

The dHCP is an open‐access project. The imaging and demographic data used in this study can be downloaded by registering at https://data.developingconnectome.org/.
