# Supplementary material for: Effects of gestational age at birth on perinatal structural brain development in healthy term‐born babies
Source: Hum Brain Mapp. 2021 Dec 12;43(5):1577–89. doi: 10.1002/hbm.25743 (PMC8886657; doi:10.1002/hbm.25743)
Supplement: Supplementary file 1 — Effects of gestational age at birth on perinatal structural brain development in healthy term born babies: Supplementary Material [file HBM-43-1577-s001.pdf]

Effects of gestational age at birth on perinatal structural brain development in healthy term-born babies: Supplementary Material

Oliver Gale-Grant[1,2,3], Sunniva Fenn-Moltu[1,2], Lucas G.S. França[1,2], Ralica Dimitrova[1,2], Daan Christiaens[2,4], Lucilio Cordero-Grande[2,5], Andrew Chew[2], Shona Falconer[2], Nicholas Harper[2], Anthony N Price[2], Jana Hutter[2], Emer Hughes[2], Jonathan O'Muircheartaigh[1,2,3], Mary Rutherford[2], Serena J Counsell[2], Daniel Rueckert[6,7], Chiara Nosarti[2,8], Joseph V Hajnal[2], Grainne McAlonan[1,3], Tomoki Arichi[2,9,10], A David Edwards[2,3]*, Dafnis Batalle[1,2]†*

1. Department of Forensic and Neurodevelopmental Science, Institute of Psychiatry, Psychology & Neuroscience, King's College London, United Kingdom
2. Centre for the Developing Brain, School of Imaging Sciences & Biomedical Engineering, King's College London, United Kingdom
3. MRC Centre for Neurodevelopmental Disorders, King's College London, United Kingdom
4. Department of Electrical Engineering, ESAT/PSI, KU Leuven, Leuven, Belgium
5. Biomedical Image Technologies, ETSI Telecomunicación, Universidad Politécnica de Madrid & CIBER-BBN, Madrid, Spain
6. Department of Computing, Imperial College London, United Kingdom
7. Department of Medicine and Informatics, Technical University of Munich, Munich, Germany
8. Department of Child & Adolescent Psychiatry, Institute of Psychiatry, Psychology & Neuroscience, King's College London, United Kingdom
9. Paediatric Neurosciences, Evelina London Children's Hospital, Guy's and St Thomas' NHS Foundation Trust, United Kingdom
10. Department of Bioengineering, Imperial College London, United Kingdom

*Contributed equally

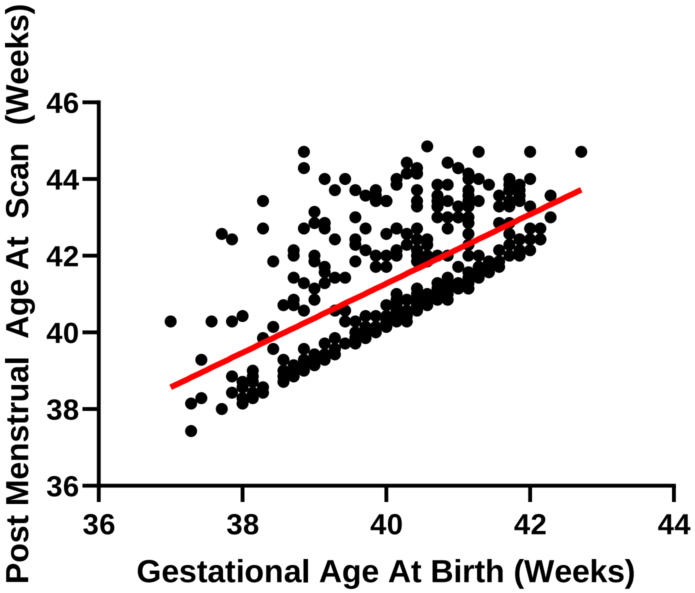

Supplementary Figure 1 – Association of Gestational Age at Birth with Post Menstrual Age at Scan (n=480). r^2=0.40, p<0.0001

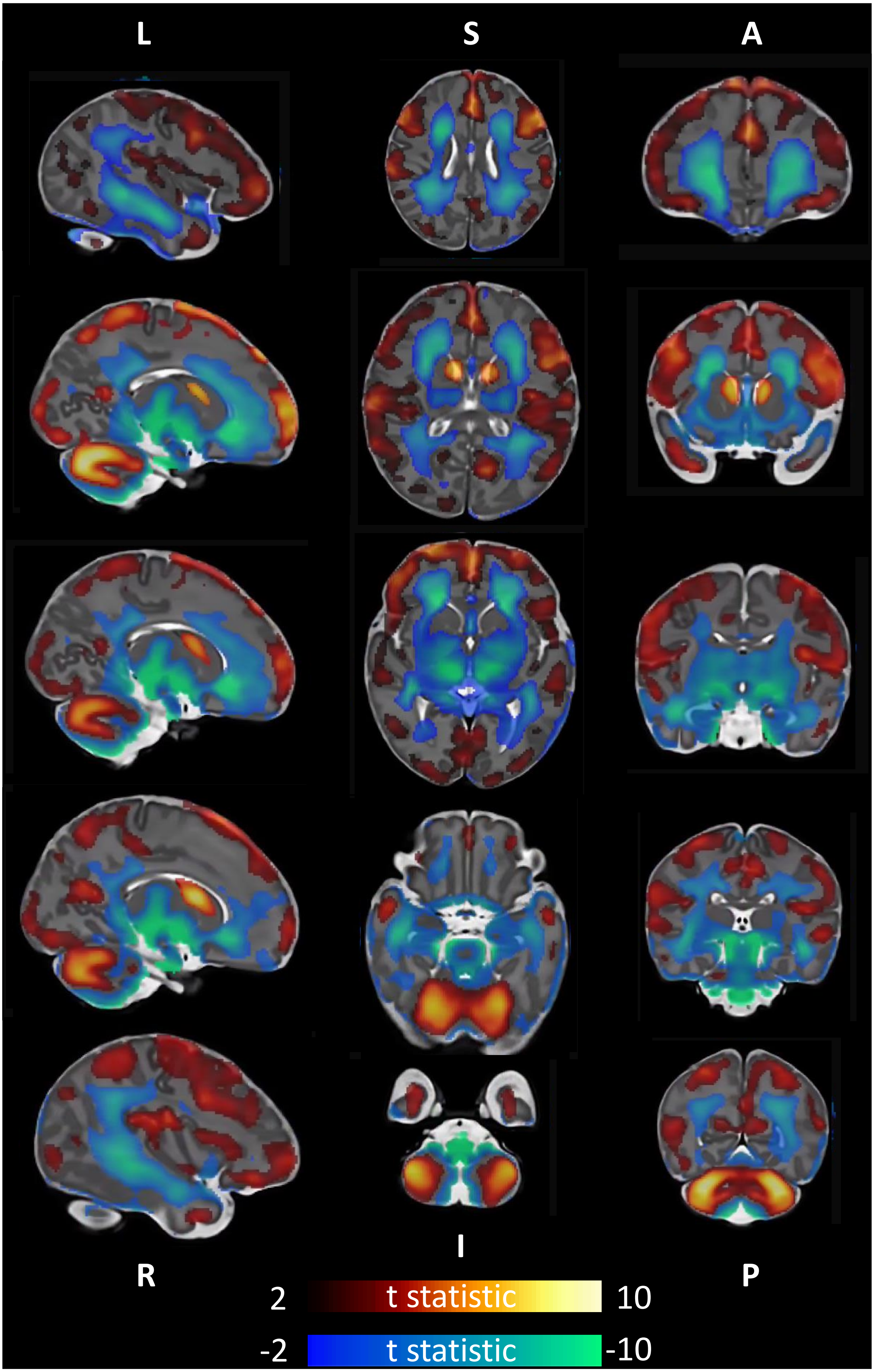

***Supplementary Figure 2** – Association of post menstrual age at scan with brain volume*. T statistic of areas of significant (p<0.025) correlation shown - positive in red-yellow, negative in blue-green. 1st column left to right, 2nd column superior to inferior, 3rd column anterior to posterior. Analysis corrected for sex.

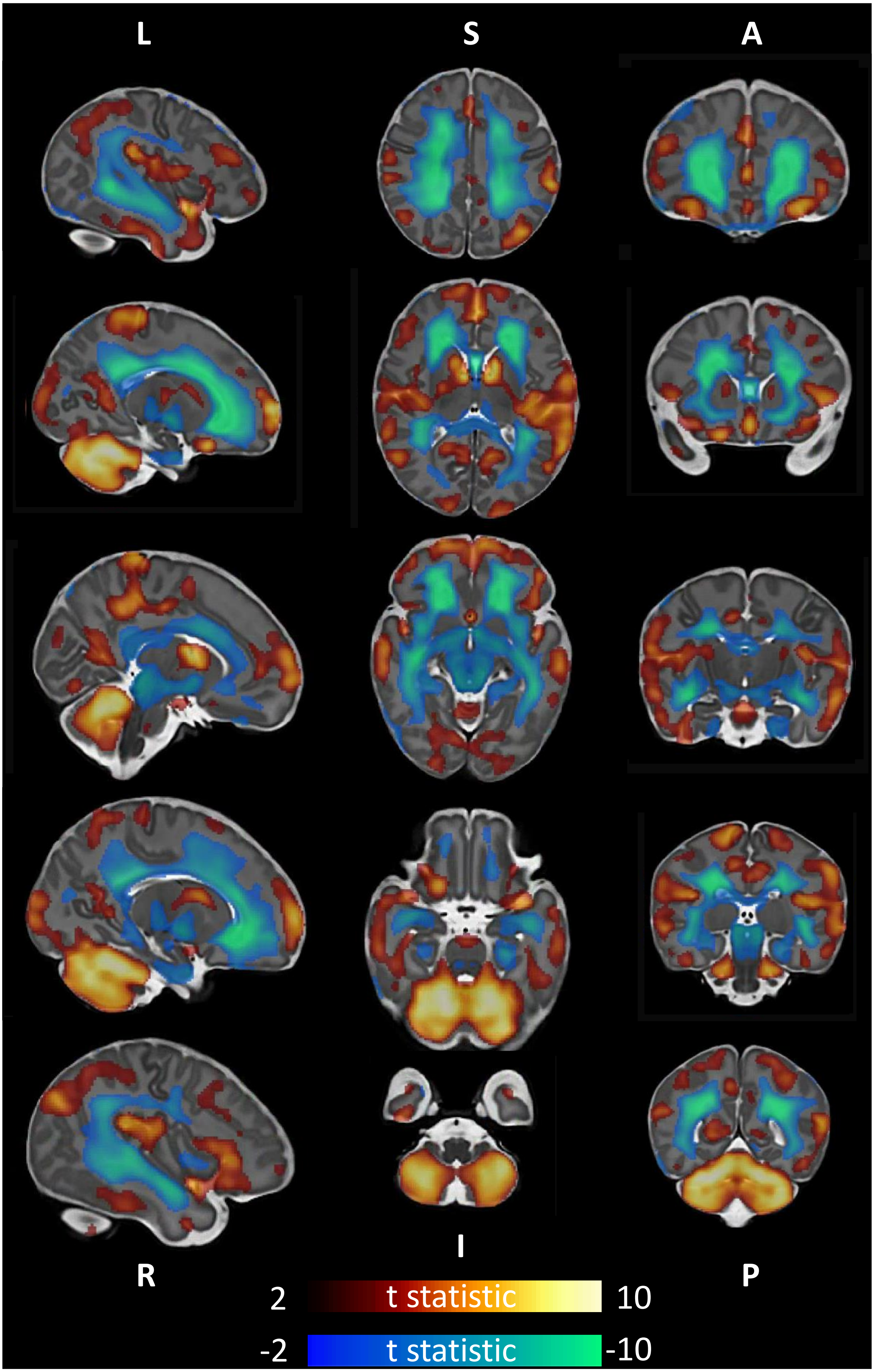

***Supplementary Figure 3** – Association of gestational age at birth with brain volume, uncorrected for post menstrual age at scan*. T statistic of areas of significant ($p<0.025$) correlation shown - positive in red-yellow, negative in blue-green. 1st column left to right, 2nd column superior to inferior, 3rd column anterior to posterior. Analysis corrected for post natal age (days) and sex.

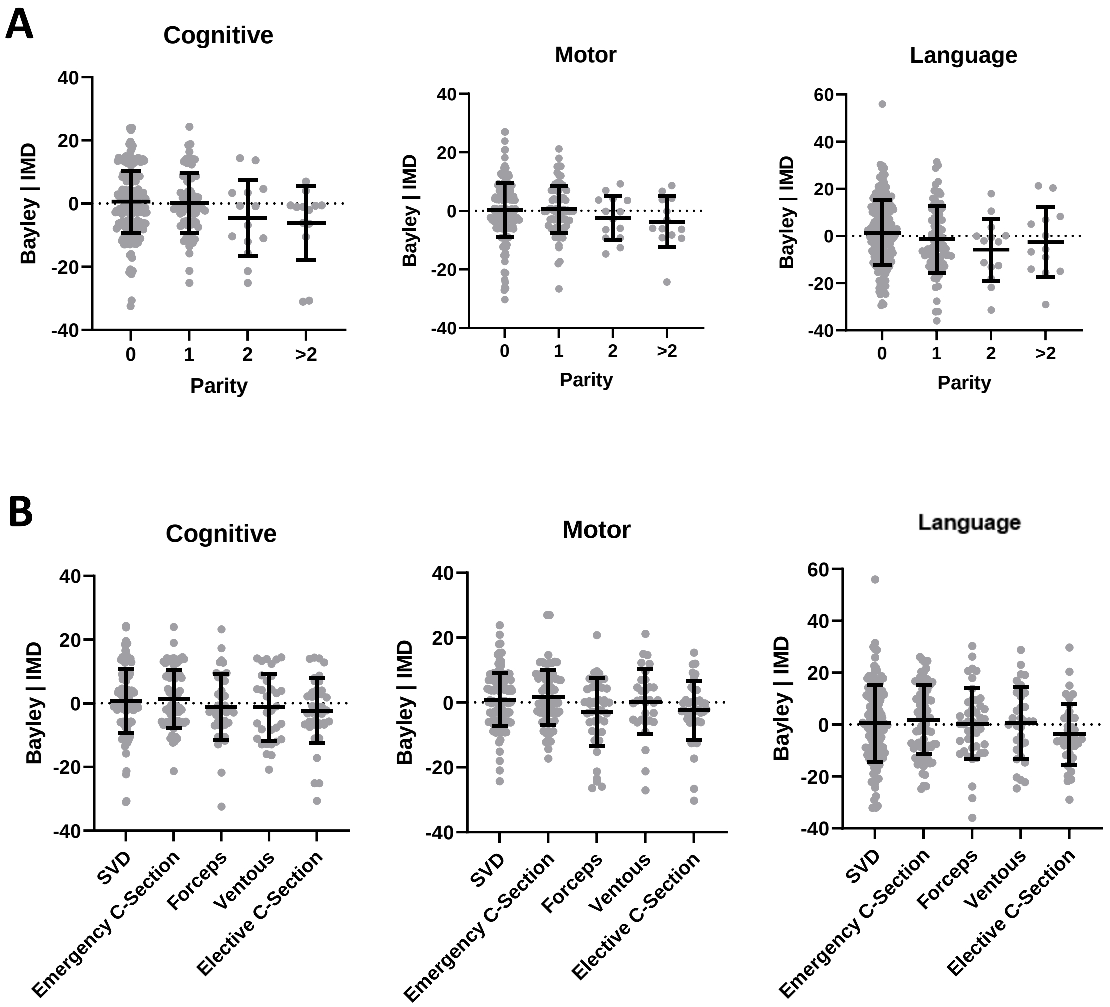

Supplementary Figure 4 – *Associations of parity* ***(A)*** *and delivery method* ***(B)*** *with Bayley-III Composite Scores (adjusted for Index of Multiple Deprivation score). Differences between groups assessed by ANOVA. No significant results seen.*

Supplementary Table 1 - *Association of regional volumes (n=332) and diffusion measures (n=281) with neurodevelopment at 18 months. Regional volumes positively and negatively associated with gestational age at birth (shown in Figure 1), and white matter regions associated with gestational age at birth (shown in Figure 2) are adjusted for postmenstrual age at scan and sex prior, and Bayley-III scales are corrected for Index of Multiple Deprivation score. Associations assessed by linear regression. The two significant associations are shown in more detail in Figure 4.*

	**Cognitive**		**Motor**		**Language**	
	r^2	p	r^2	p	r^2	p
**Volume (Positive Correlation with GAAB)**	0.004	0.226	0.008	0.092	0.008	0.757
**Volume (Negative Correlation with GAAB)**	0.016	0.021*	0.044	<0.001***	0.001	0.146
**FA**	0.001	0.694	0.001	0.582	0.003	0.384
**MD**	0.002	0.426	0.000	0.949	0.005	0.213
**AD**	0.001	0.583	0.003	0.333	0.002	0.430
**RD**	0.002	0.471	0.000	0.874	0.005	0.219
